# Book Notices

**Published:** 1855-12

**Authors:** 


Professor Andrews.

FROM an article in the *Peninsular Journal of Medicine* we extract the following just tribute to Prof. Andrews, now lecturer on Comparative and Demonstrator of Human Anatomy in Rush Medical College:

His successors to the editorial chair of the *Peninsular Journal* say:

"The vacation by Professor Andrews, of the chair of Comparative Anatomy, in the University of Michigan, and his subsequent removal from the State, devolve upon the undersigned the necessity of assuming the editorial charge of the Peninsular Medical Journal, which was called into existence by his enterprise, and sustained during the first year of its existence, solely by his energy and ability. Although the editorial force has been, since that time, numerically quadrupled, we do not expect to enter upon the management of it with a more exalted purpose in view, or to manifest a warmer zeal in the cause to which it is devoted, or to exhibit a higher order of talent than it was the good fortune of our worthy predecessor to possess, and employ in its service.

We reiterate Professor Palmer's expressions of good-will to his retiring colleague, because of the pleasure it affords us to do so; and take this occasion to say to his new associates, that few men possess a clearer intellect, and none a truer heart than they will find in the bosom of the alumnus they have snatched from the University of Michigan. The benediction of his alma mater follows him.

History of Medicine from its origin to the nineteenth century, with an appendix containing a philosophical and historical review of medicine to the present time By P. V. RENOURD, M. D., translated from the French by CORNELIUS G. COMEGYS, M. D., professor of the Institutes of Medicine, Miami Medical College. Cincinnati: Moore, Wilstach, Keys & Co. New York: Miller, Orton, & Mulligan. Boston: Whittemore, Mills & Hall. Philadelphia: J. B. Lippincott & Co. 1856.

OUR thanks are due both to the publishers and translator for this beautiful edition of Renourds history of medicine. If we are not much mistaken it will be very generally sought after to beguile the intervals of severe study and severer labor of professional life.

The design of the work will be understood from the following extract:

"I divide into three Books, or three Ages, all past time. The First Age commences with the infancy of society, as far back as historic tradition carries us, and terminates toward the end of the second centuryof the Christian era, at the death of Galen, during the reign of Septimus Severus. This lapse of time constitutes, in Medicine, the Foundation age. The germ of the Healing Art, concealed, at first, in the instincts of men, is gradually developed the basis of the science is laid, and great principles are discussed. The human mind, always impatient, surpasses in its speculations, the limitsof the known and possible. Many branches of the art, such as symptomatology and Prognosis, are carried to a remarkable degree of perfection.

The Second Age, which may be called the Age of Transition, offers very little material to the history of medicine. We see no longer the conflicts and discussions between partisans of different doctrines; the medical sects are confounded. The art remains stationary, or imperceptibly retrogrades. I can not better depict this epoch than by comparing it to the life of an insect in the nympha state; though no interior change appears, an admirable metamorphosis is going on, imperceptibly, within. The eye of manonly perceives the wonder after it has been finished.

Thus, from the fifteenth century, which is the beginning of the third and last Agê of Medicine, or the Age of Renovation, Europe offers us a spectacle of which the most glorious eras of the republics of Greece and Rome only gives us an ieda. It would seem as if a new life was infused into the veins of the inhabitants of this part of the world; the sciences, fine arts, industry, religion, social institutions, all are changed. A multitude of schools are opened fôr teaching Medicine. Establishments which had no models among the ancients, are created for the purpose of extending to the poorer classes the benefits of the Healing Art. The ingenious activity of modern Christians explores and is sufficient for everything.

These three grand chronological divisions do not suffice to classify, in our minds, the principal phases of the history of Medicine; consequently, I have subdivided each age into a smaller number of sections, easy to be retained, and which I have named

Periods. The first Age embraces four periods, the second and third ages each, two.

I will now indicate succinctly, each of these secondary divisions, without attempting, at present, to justify them, for this will be done in its proper place in the course of the work.

The first period, which we name *Primitive Period*, or that of *Instinct*, ends with the ruin of Troy, about twelve centuries before the Christian era.

The second, called the *Mystic* or *Sacred Period*, extends from the dissolution of the "Pythagorean Society" to abut the year 500, A. C.

The third period, which ends at the foundation of the Alexandria Library, A. C., 320, we name the *Philosophic Period.*

The Fourth, which we designate the *Anatomic*, extends to the end of the first age, i. e., to the year 200 of the Christian era.

The fifth is called the *Greek Period*; it ends at the destruction of the Alexandrian Library, A. D. 640

The sixth receives the surname of *Arabic*, and closes with the fourteenth century.

The seventh period, which begins the third age, comprises the fifteenth and sixteenth centuries: it is distinguished as the *Frudite.*

Finally, the eight, or last period, embraces the seventeenth and eighteenth centuries. I call it the *Reform Period.*

From the history of the past we are to learn wisdom for the present, and gain light for the future. Renourd's work has therefore a higher and more permanent use than simply to amuse or relieve the tedium of a passing hour. We wish it could be read by every physician in the land. It would make him think better of his profession, both as an art and a science, and give him clearer and higher views of the duty which he owes it and to the community in which he lives.

The work is for sale by D. B. Cook & Co , Chicago, Ill.

